# ﻿Molecular characterisation of three *Ixodes* (*Pholeoixodes*) species (Ixodida, Ixodidae) and the first record of *Ixodes* (*Pholeoixodes*) *kaiseri* from Slovakia

**DOI:** 10.3897/zookeys.1158.101936

**Published:** 2023-04-21

**Authors:** Zuzana Krumpálová, Barbara Mangová, Slávka Purgatová, Yuliya M. Didyk, Mária Kazimírová

**Affiliations:** 1 Constantine the Philosopher University, Faculty of Natural Sciences and Informatics, Tr. A. Hlinku 1, Nitra, Slovakia Constantine the Philosopher University Nitra Slovakia; 2 Institute of Zoology, Slovak Academy of Sciences, Dúbravská cesta 9, Bratislava, Slovakia Institute of Zoology, Slovak Academy of Sciences Bratislava Slovakia; 3 I.I. Schmalhausen Institute of Zoology, National Academy of Sciences of Ukraine, Vul. B. Khmelnytskogo 15, Kyiv, Ukraine I.I. Schmalhausen Institute of Zoology, National Academy of Sciences of Ukraine Kyiv Ukraine

**Keywords:** European badger, hedgehog, *Ixodescanisuga*, *Ixodeskaiseri*, red fox, ticks

## Abstract

A study of ticks on wildlife was carried out in the area of Levice, Bratislava, Stupava, and Vrbovce (south-western Slovakia) during 2021 and 2022. Overall, 512 ticks were collected from 51 individuals of six wild mammalian species. Eight tick species were identified, namely *Dermacentorreticulatus*, *D.marginatus*, *Haemaphysalisinermis*, *H.concinna*, *Ixodesricinus*, *I.hexagonus*, and two *Ixodes* spp. *Ixodeshexagonus* were collected from northern white-breasted hedgehogs (*Erinaceusroumanicus*), females belonging to *Ixodes* spp. were collected from red fox (*Vulpesvulpes*) and nymphs from European badger (*Melesmeles*). *Ixodeshexagonus* and the *Ixodes* spp. were identified morphologically and molecularly based on sequences of fragments of two mitochondrial genes, *COI* and 16S rRNA. Molecular analysis of *Ixodes* spp. confirmed the identity of *Ixodeskaiseri* Arthur, 1957 and *I.canisuga* (Johnston, 1849). Sequence analyses show that the *I.kaiseri* isolate from Slovakia is identical to *I.kaiseri* isolates from Romania, Poland, Germany, Turkey, and Croatia. We demonstrate for the first time the presence of *I.kaiseri* in Slovakia using both morphological and molecular methods.

## ﻿Introduction

Ticks (Ixodida) belong to the most important ectoparasites of terrestrial vertebrate species. Hard ticks of the subgenus Pholeoixodes Schulze, 1942 (*Ixodes*, Ixodidae) are usually associated with burrow-dwelling mammals and terrestrial birds that nest in cavities (tree holes or burrows). All representatives of the subgenus Pholeoixodes are endophilic three-host ticks with a rather uniform circle of hosts for all active ontogenetic stages. For a long time, the taxonomy of the subgenus Pholeoixodes was rather confused (Estrada-Peña et al. 2017a; Tsapko 2018). This can be explained by the large intraspecific morphological variability of the species, when morphologically differing specimens of the same species were described under different names or were misdiagnosed. At the same time, as mentioned by Filippova (1977) and Emelyanova (1979), new findings from previously unknown hosts were described as new species. In the Western Palaearctic the Ixodes (Pholeoixodes) group currently includes I. (P.) kaiseri Arthur, 1957, I. (P.) crenulatus Koch, 1844, I. (P.) canisuga Johnston, 1849, I. (P.) hexagonus Leach, 1815, and I. (P.) rugicollis Schulze & Schlottke, 1929, which usually feed on mammals, particularly carnivores (mainly Canidae and Mustelidae) and hedgehogs (Erinaceidae), and I. (P.) arboricola Schulze & Schlottke, 1929 and I. (P.) lividus Koch, 1844, which parasitize birds (Karbowiak et al. 2017; Estrada-Peña et al. 2017a; Guglielmone et al. 2020). However, the taxonomic status of *I.crenulatus* and *I.canisuga* needs further clarification (Estrada-Peña et al. 2017b; Guglielmone et al. 2020; Karbowiak et al. 2020).

*Ixodeskaiseri* was for the first time described from Common Egyptian fox from Burg EJ Arab, Mariut, Western Desert Governorate, Egypt (Arthur 1957). It is a Palaearctic species and all its parasitic stages have been found on Carnivora (Canidae, Felidae, and Mustelidae) and Erinaceomorpha (Erinaceidae). Adults were also recovered from Rodentia (Sciuridae), adults and nymphs were collected from Carnivora (Hyaenidae) and Rodentia (Hystricidae), and larvae and nymphs from Rodentia (Cricetidae) (Guglielmone et al. 2020). There are no records of *I.kaiseri* parasitizing humans. The presence of *I.kaiseri* has been reported in several European countries including Poland, Germany, Hungary, Serbia, Croatia, Romania, Ukraine, Malta (Akimov and Nebogatkin 2002; Hornok et al. 2017, 2020a, 2021; Dwużnik et al. 2020; Krčmar et al. 2022), and Turkey (Orkun and Karaer 2018). Hornok et al. (2017), while reporting presence of *I.kaiseri* specimens on red foxes and dogs in Romania, also noted that the species is more widespread in Europe than previously thought. The species was also recorded in Moldova, southern Ukraine, Georgia, Azerbaijan, Iran, Syria, Lebanon, and Israel (Filippova 1977; Tsapko 2018). In addition, it is known from Kazakhstan (Dzungarian Alatau) and from the North Caucasus (Tsapko 2018). In most parts of the established range, *I.kaiseri* co-occurs with the closely related *I.crenulatus* (*I.canisuga*?) and can also simultaneously parasitize the same host individuals (Tsapko 2018). Predatory mammals play the primary role as hosts of this tick species. According to Filippova (1977), *I.kaiseri* was found on the following hosts: European badger *Melesmeles*, red fox *Vulpesvulpes*, raccoon dog *Nyctereutesprocyonoides*, domestic dog *Canislupusfamiliaris*, steppe polecat *Mustelaeversmanii*, striped hyena *Hyaenahyaena*, wildcat *Felissilvestris*, and jungle cat *F.chaus*. In addition to carnivores, *I.kaiseri* were collected from the Indian crested porcupine *Hystrixindica* and from the northern (*Erinaceusroumanicus*) and southern (*E.concolor*) white-breasted hedgehogs. Hornok et al. (2017) analysed the phylogenetic relationships by using two mitochondrial genes and morphological differences in females of three Ixodes species of the subgenus Pholeoixodes including *I.kaiseri*. Furthermore, Hornok et al. (2021) published pictorial keys for identification of *I.kaiseri*, *I.canisuga*, and *I.hexagonus* males, nymphs, and larvae. *Ixodeskaiseri* was found to harbour several tick-borne pathogens of veterinary and medical importance (Hornok et al. 2020b; Wodecka et al. 2022); however, it is not clear if and to what extent this species contributes to pathogen circulation in nature.

*Ixodescanisuga*, considered by some authors as a synonym of *I.crenulatus* (e.g. Filippova and Uspenskaya 1973; Filippova 1977; Siuda 1993), is associated with mammals which inhabit burrows. The most infested species are medium-sized mustelids and canids such as the red fox and European badger, among others (Cornely and Schultz 1992; Santos-Silva et al. 2011). This tick species is also a common parasite of domestic dogs and has also been found on cats (Liebisch and Walter 1986; Földvári and Farkas 2005; Hornok et al. 2017). Overall, *I.canisuga* is distributed from the Spanish Pyrenees through Russia, Iran, Afghanistan, India (Kashmir), to eastern China (Estrada-Peña et al. 2017a). In Europe, this species has been recorded in almost all countries, from United Kingdom, Ireland, France, Austria, and Germany in the west (Walter et al. 1986; Cornely and Schultz 1992; Ogden et al. 2000), Portugal in the south (Santos-Silva et al. 2011), and in countries of central and south-eastern Europe, i.e., Hungary, Poland, Romania, Croatia, Serbia, and Bosnia and Herzegovina (Hornok et al. 2017; Krčmar et al. 2022). It was possibly recorded also in the former Czechoslovakia, but under different names (Černý 1972).

*Ixodeshexagonus* is a common species in the Western Palaearctic. However, the species was sometimes mistakenly identified as *I.canisuga* (Hornok et al. 2017). *Ixodeshexagonus* is associated with hedgehogs, *Erinaceuseuropaeus* and *E.roumanicus*, which are the main hosts among the broad spectrum of medium-sized burrow-inhabiting mammals. The species was also found on wild carnivores such as red foxes, mustelids, and the European badger (Estrada-Peña et al. 2017a; Karbowiak et al. 2020). In addition, *I.hexagonus* can frequently be encountered on domestic pets (cats and dogs) and harbours various tick-borne pathogens (Walker 2018). The distribution of *I.hexagonus* covers almost the whole of Europe, ranging from the British Islands in the west, across all the countries in central and eastern Europe, including Slovakia (Černý 1972; Estrada-Peña et al. 2017a; Karbowiak et al. 2020). The species was also found outside this range, e.g. in north-western Iran (Tavassoli and Mohamadi 2015).

## ﻿Materials and methods

### ﻿Ethics statement

The study complies with current laws of the Slovak Republic and with species conservation guidelines. The animals were killed for hunting reasons during the legal hunting season and not specifically for this study. Collections of ticks from hedgehogs were in accordance with Decision No. 8711/2022-6.3 – Exemption from Act No. 543/2022 on Nature and Landscape Protection.

### ﻿Study area

Hunted animals originated from the area of Žemberovce (south-western Slovakia). The village of Žemberovce (48°15'30"N, 18°44'30"E) borders with the town of Levice. There are extensive thermophilous forest communities, mainly oak–hornbeam Carpathian forests with *Carpinusbetulus* and *Quercuspetraea*. Mixed oak forests (*Quercuscerris*, *Acercampestre*, *Cerasusavium*, and *Tiliacordata*) are also found in suitable habitats. Hedgehogs were captured in parks within residential zones of Bratislava (48°14'85"N, 17°10'77"E), Stupava (48°27'89"N, 16°99'55"E), and in the village of Vrbovce (48°79'98"N, 17°46'89"E) (western Slovakia).

### ﻿Tick collection and identification

We collected ticks individually with tweezers directly from the skin of hunted mammals. The searches for hedgehogs were performed at night (10 p.m. to 3 a.m.) by two persons. The equipment consisted of headlamps and thick welding gloves for hedgehog handling. After removing the ticks, all the hedgehogs were released back in their original capture location without significant manipulation.

Ticks were stored in 80% ethanol at 4 °C. They were examined morphologically; adult ixodid ticks are usually easier to identify to species than immature stages, and therefore morphological comparisons followed Siuda (1993), Slovák (2010, 2014), and Bristol University Tick ID (http://www.bristoluniversitytickid.uk). Species of the subgenus Pholeoixodes are the most problematic group in the genus *Ixodes*. Reports of *Pholeoixodes* spp. from carnivores are frequently contradictory, and their identification is not based on key diagnostic characters. Moreover, identification of engorged ticks is even more difficult. Nymphs of *Ixodes* spp. were morphologically identified according to Hornok et al. (2017, 2021) using a stereomicroscope (Olympus SZ61). Photographs were taken using a Leica M205C stereo microscope and a Leica Flexacam C1 camera, including LAS X software with a Z-stack projection tool. The photographed specimens were immersed in 80% ethanol, because they were stored for further molecular detections of microorganisms.

### ﻿Molecular analysis

Genomic DNA was isolated individually from legs of females and nymphs of *I.hexagonus* and *Ixodes* spp. by the method of alkaline hydrolysis with modifications (Guy and Stanek 1991) and from whole engorged ticks with the DNeasy Blood & Tissue Kit (Qiagen, Hilden, Germany) following the manufacturer’s instructions. DNA samples were stored at −20 °C until further analyses. Fragments of the mitochondrial 16S rRNA and the *COI* genes were chosen for molecular analyses and tick species identification (Black and Piesman 1994; Hornok et al. 2017). PCR amplified approximately 710 bp of the *COI* gene using the primers LCO1490 and HCO2198 (Hornok et al. 2017) and approximately 460 bp of the 16S rRNA gene of Ixodidae, using the primers 16S+1 and 16S-1 (Black and Piesman 1994). PCR products were analysed by electrophoresis in 1.5% agarose gel stained with GoodView Nucleic Acid Stain (SBS Genetech, Beijing, China) and visualized under UV light. Amplicons were purified using a QIAquick Spin PCR Purification Kit (Qiagen, Hilden, Germany) as described by the manufacturer. The sequencings were performed by Eurofins Genomics Europe (https://www.eurofinsgenomics.eu). DNA sequences were compared with available databases in GenBank using the Basic Local Alignment Search Tool (BLAST) NCBI. The MEGA model selection method was applied to choose the appropriate model for phylogenetic analyses. Phylogenetic analyses were conducted using the maximum likelihood method based on the Tamura-Nei model (Tamura and Nei 1993) using MEGA v. 7.0 (Kumar et al. 2016). Nucleotide sequences of *COI* genes obtained in this study were submitted to BOLD: The Barcode of Life Data System (http://www.barcodinglife.org) under the accession numbers UZINS193 23 SK, UZINS194 23 SK, UZINS195 23 SK, UZINS197 23 SK, UZINS198 23 SK, UZINS199 23 SK, UZINS204 23 SK, UZINS205 23 SK, UZINS206 23 SK, and UZINS182 23 SK.

### ﻿Data availability statement

The data presented in this study are available upon request from the corresponding author. Nucleotide sequences of *COI* genes derived from the study are available in BOLD: The Barcode of Life Data System (http://www.barcodinglife.org).

## ﻿Results

During 2021 and 2022, we obtained ticks from hunted wild mammals belonging to five species in the Levice region of south-western Slovakia. The wildlife consisted of red fox *Vulpesvulpes*, European badger *Melesmeles*, wild boar *Susscrofa*, red deer *Cervuselaphus*, and European roe deer *Capreoluscapreolus*. At the same time, we collected ticks from hedgehogs (*Erinaceusroumanicus*) in green areas of Bratislava, Stupava, and Vrbovce.

In total, 335 ticks (54 nymphs, 191 females, and 90 males) were collected from 35 hunted wild mammalian individuals and 178 ticks (48 larvae, 77 nymphs, 44 females, and 9 males) from hedgehogs. We identified the presence of eight tick species, namely *Dermacentorreticulatus*, *D.marginatus*, *Haemaphysalisinermis*, *H.concinna*, *Ixodesricinus*, *I.canisuga*, *I.hexagonus*, and *I.kaiseri*.

In terms of species (Table 1), *I.ricinus* was the most frequent and abundant species, accounting for 71.1% of all specimens collected, and it was found on five hosts (wild boar, red deer, roe deer, red fox, and hedgehog). *Dermacentorreticulatus* accounted for 7% of all specimens and *D.marginatus* for 5.1%; both were confirmed on one host, wild boar. Fewer *H.concinna* (4.3%) and *H.inermis* (5.9%) were found on two hosts (wild boar and red deer), and *I.hexagonus* (4.5%) was found on hedgehogs only. The remaining specimens (2.2%) belonged to *Ixodes* spp. and were collected from red fox and European badger.

**Table 1.** Total number of ticks collected from wildlife (all life stages combined, identification comprises also results of molecular analyses).

Host/tick species	*I.ricinus*	*I.canisuga*	*I.kaiseri*	*I.hexagonus*	*D.reticulatus*	*D.marginatus*	*H.inermis*	*H.concinna*
*Vulpesvulpes*	2		2					
*Melesmeles*		5	4					
*Capreoluscapreolus*	19							
*Cervuselaphus*	146						3	7
*Susscrofa*	43				36	26	27	15
*Erinaceusroumanicus*	155			23				
Total	365	5	6	23	36	26	30	22

### ﻿Morphological diagnosis

**Figure 1.**
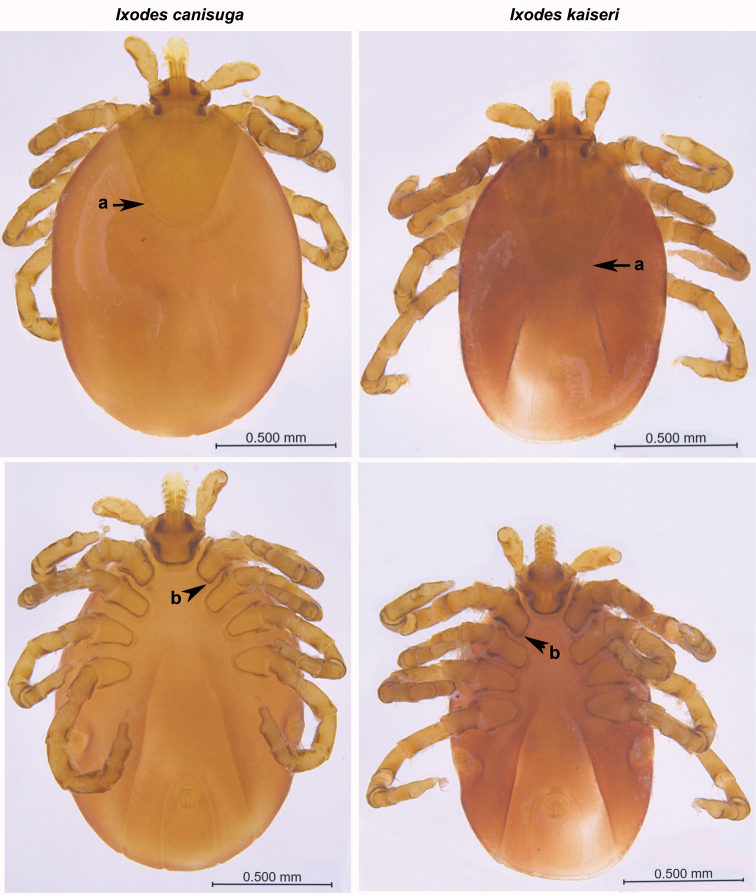
Partially engorged *Ixodescanisuga* and *I.kaiseri* nymphs (dorsal views – upper panels, ventral views – lower panels) collected from *Melesmeles* in Slovakia. Differences are seen in the shape of scutum (**a**) which is shorter in *I.canisuga* than in *I.kaiseri*. There is a broad internal spur on coxa I (**b**) in both species. Photo: Ľ. Vidlička

**Figure 2.**
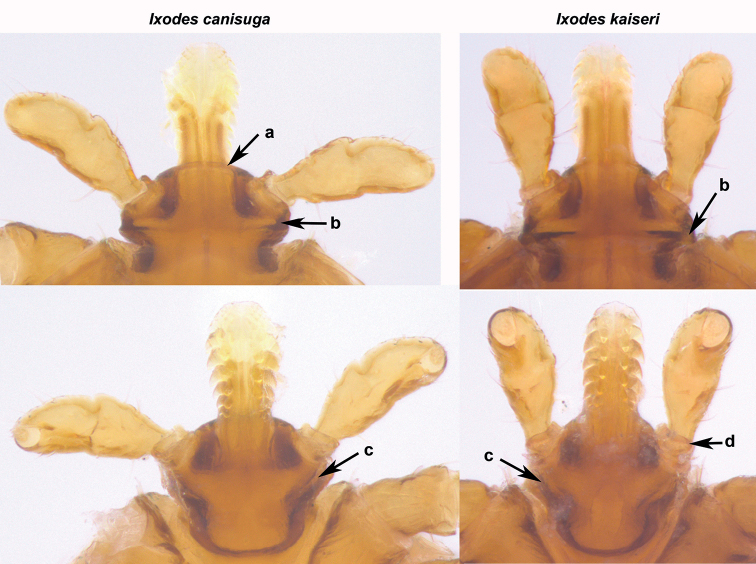
Gnathosoma of *Ixodescanisuga* and *I.kaiseri* nymphs (dorsal views – upper panels, ventral views – lower panels) collected from *Melesmeles* in Slovakia. *Ixodescanisuga* – anteriorly flattened basis capituli (**a**); dorsally absence of cornua (**b**); long, moderately thick auricular ridge (**c**). *Ixodeskaiseri*: cornua well developed (**b**); short, thin auricular ridge (**c**); anteriorly rounded, sclerotized protuberance on palpal segment I (**d**).

In total, three female ticks and one male were collected from red fox and nine nymphs from European badger. Initially, based on the existence of an anal groove and the typical structure of the mouthparts, they were morphologically identified as *Ixodes* spp. One of the females and the male from red fox were further identified as *I.ricinus* and the other two females from the red fox and all nymphs from the European badger as *Ixodes* spp. Because mouthparts of the females and part of the nymphs were damaged during their removal from the host skin, morphological identification was only possible in six undamaged nymphs. Two of them were identified as *I.canisuga* and four as *I.kaiseri* (Figs 1, 2) according to detailed morphological examinations showing that the nymphs have the characters described by Hornok et al. (2021).

### ﻿Molecular identification

By amplification of the *COI* gene, two *Ixodes* spp. females from the red fox and four nymphs from the European badger were identified as *I.kaiseri* and four nymphs as *I.canisuga*. Amplification was not successful for one nymph morphologically identified as *I.canisuga*. By amplification of fragments of the 16S rRNA gene in DNA samples from one female and two nymphs of *I.kaiseri* and three nymphs of *I.canisuga* the identification of the species was confirmed. Molecular analyses of amplified fragments of the *COI* and 16S rRNA genes in DNA samples derived from two females and one nymph of *I.hexagonus* from hedgehogs confirmed the correct identification of the species based on morphology.

**Figure 3.**
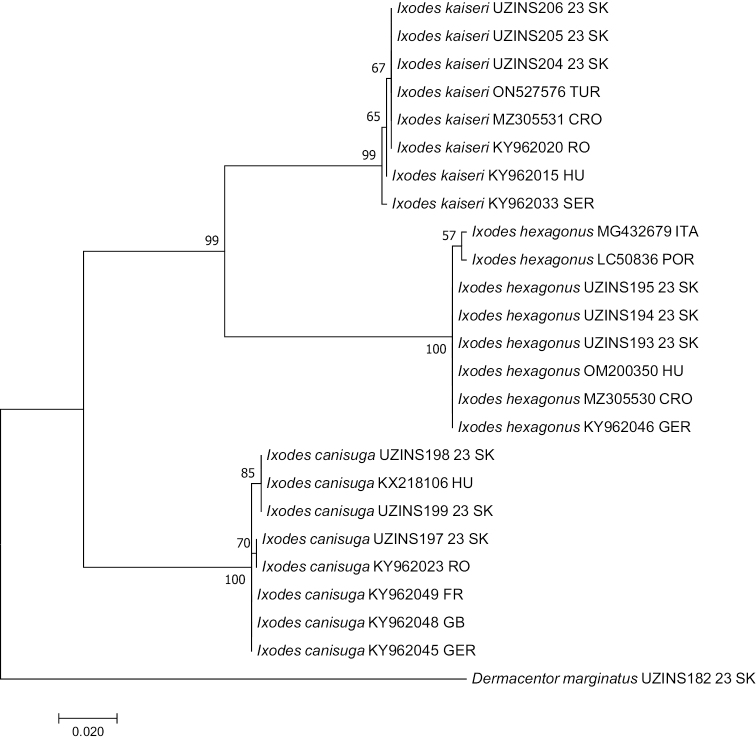
Phylogenetic tree of *Ixodes* spp. constructed by using a maximum-likelihood analysis of the *COI* gene. Bootstrap 1000 bp. Branch lengths represent the number of substitutions per site inferred according to the scale shown. *COI* gene of *D.marginatus* (iBOL source) was used as the outgroup.

The isolates UZINS204 23 SK, UZINS205 23 SK, and UZINS206 23 SK were uniform based on the *COI* sequences. BLAST search showed 100% identity of the Slovak isolates with *COI* gene sequences of *I.kaiseri* isolates from Turkey (ON527576), Croatia (MZ305531), and Romania (KY962020), 99.84% identity with isolate from Hungary (KY962015), and 99.52% with isolate from Serbia (KY962033). Isolates UZINS193 23 SK, UZINS194 23 SK, and UZINS195 were uniform and showed 100% identity with the *COI* gene sequences in isolates of *I.hexagonus* from Hungary (OM200350), Croatia (MZ305530), and Germany (KY962046), and 99.54% identity with isolates from Italy (MG432679) and Portugal (LC508366). Isolate UZINS197 23 SK showed 100% identity with the *COI* gene sequence of *I.canisuga* isolate from Romania (KY962023), and 99.84% identity with isolates from France (KY962049), United Kingdom (KY962048), Germany (KY962045), and 99.53% with isolate from Hungary (KX218106). Samples UZINS198 23 SK and UZINS199 23 SK showed 100% identity with the *COI* gene sequences of *I.canisuga* isolate from Hungary (KX218106) and 99.68% with isolates from France (KY962049), United Kingdom (KY962048), and Germany (KY962045) (Fig. 3).

**Figure 4.**
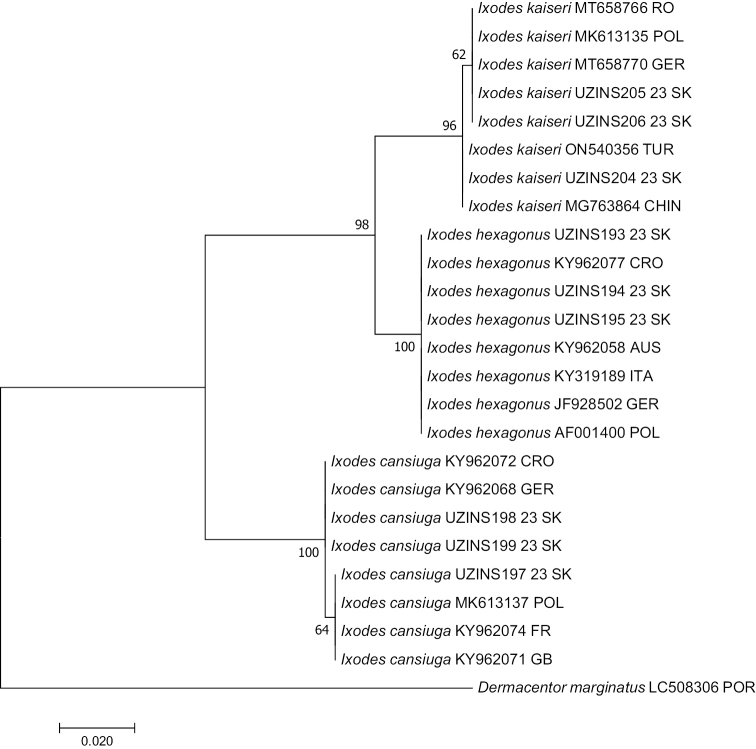
Phylogenetic tree of *Ixodes* spp. constructed by using a maximum-likelihood analysis of 16S rRNA gene. Bootstrap 1000 bp. Branch lengths represent the number of substitutions per site inferred according to the scale shown. 16S rRNA gene of *D.marginatus* (NCBI source) was used as outgroup.

Sequence of the 16S rRNA gene in our samples UZINS205 23 SK and UZINS206 23 SK showed 100% identity with the 16S rRNA gene sequences in *I.kaiseri* isolates from Poland (MK613135), Romania (MT658766), and Germany (MT658770), 99.76% identity with isolate from Turkey (ON540356), and 99.51% identity with isolate from China (MG763864). Sequence in our sample UZINS204 23 SK showed 100% identity with the 16S rRNA gene sequences in *I.kaiseri* isolate from Turkey (ON540356), 99.76% identity with isolates from Poland (MK613135) and China (MG763864), and 99.75% identity with isolates from Germany (MT658770) and Romania (MT658766). Sequences in samples UZINS193 23 SK, UZINS194 23 SK and UZINS195 23 SK were uniform and showed 100% identity with the 16S rRNA gene sequences in *I.hexagonus* isolates from Croatia (KY962077), Austria (KY962058), Germany (JF928502), and Poland (AF001400) and 99.76% identity with isolate from Italy (KY319189). Sequence of the 16S rRNA gene in our sample UZINS197 23 SK was 100% identical with the 16S rRNA gene sequences of *I.canisuga* isolate from Poland (MK613137), France (KY962074), and United Kingdom (KY962071), and 99.75% identical with isolates from Germany (KY962068) and Croatia (KY962072). Sequences in samples UZINS198 23 SK and UZINS199 23SK showed 100% identity with the 16S rRNA gene sequences in *I.canisuga* isolates from Croatia (KY962072) and Germany (KY962068), 99.76% identity with isolate from Poland (MK613137), and 99.75% with isolates from France (KY962074) and United Kingdom (KY962071) (Fig. 4).

Molecular identification with markers *COI* and 16S rRNA confirmed the morphological identification of *I.kaiseri*, *I.canisuga*, and *I.hexagonus* from Slovakia (Figs 3, 4). We demonstrated the presence of *I.kaiseri* for the first time in Slovakia using both morphological and molecular methods. Consequently, Slovakia was added to the list of countries where *I.kaiseri* was reported from red fox and European badger.

## ﻿Discussion

We recorded the co-occurrence of two endophilic Ixodes spp. of the subgenus Pholeoixodes, *I.kaiseri* and *I.canisuga* on European badger, and of *I.kaiseri* and *I.ricinus* on red fox. For the first time, the occurrence of *I.kaiseri* is confirmed in Slovakia. The presence of *I.kaiseri* in this country was predictable (Karbowiak et al. 2020), as it was found to parasitize wild carnivores and dogs in neighbouring countries of Hungary, Poland, and Ukraine, but also in Germany and south-eastern Europe (Romania, Croatia, Serbia) (Akimov and Nebogatkin 2002; Hornok et al. 2017, 2021; Dwużnik et al. 2020; Krčmar et al. 2022).

Meyer-Kayser et al. (2012) found on foxes in Germany predominance of tick larvae (48%), followed by adults (34%), and nymphs (18%). *Ixodesricinus* was the most frequent tick species, followed by *I.canisuga* and *I.hexagonus*. In previous studies from Slovakia, *D.reticulatus*, *H.concinna*, *I.ricinus*, and two *Pholeoixodes* species, *I.hexagonus* and *I.crenulatus* identified based on morphology, had been found to parasitize red foxes (Kočišová et al. 2006; Karbowiak et al. 2020). Dwużnik et al. (2020) investigated ectoparasites of red foxes in three regions of Poland. *Ixodesricinus* and *D.reticulatus* were the dominant tick species on adult foxes, but *I.kaiseri* was also recorded. We found *I.canisuga* on European badger, but not on red fox, and *I.kaiseri* on red fox and European badger. However, we examined only one individual of each host species for ticks, which is not enough to draw conclusions on their parasitofauna in Slovakia.

*Ixodesricinus* and *I.hexagonus* are common ectoparasites of hedgehogs in urban and suburban areas of Europe. For example, by examining hedgehogs in a city park in Budapest (Hungary), the high prevalence (93.7%) of *I.ricinus* and presence of *I.hexagonus* were recorded. Nymphs prevailed in both species (Földvári et al. 2011). In four urban habitats in Cluj-Napoca city, Romania, in addition to birds and small mammals, ticks were collected from northern white-breasted hedgehogs. *Ixodesricinus* prevailed (89.7%), followed by *I.hexagonus* (7.7%) and *Haemaphysalispunctata* (2.6%). With regards to life stages, larvae dominated in *I.ricinus* (67%) and *H.punctata* (71.4%) and females (75.9%) in *I.hexagonus* (Borşan et al. 2020). We identified *I.ricinus* and *I.hexagonus* on hedgehogs from three urbanized areas of Slovakia. Immature stages (nymphs and larvae) prevailed in both species. Thus, the ratios of adult ticks and immature stages differ between sites and are probably affected by microclimate and the presence of other hosts for ticks.

In general, changes in land usage patterns and climate, i.e., milder winters and earlier onset of spring in the northern hemisphere, can significantly affect the geographic distribution, phenology, and population density of some tick species and the occurrence of tick-borne zoonoses (Gray et al. 2009; Gilbert 2021). In addition, ticks can easily spread and colonize new regions via international pet trade, domestic animal transport, or bird migration (Földvári et al. 2016). Given the vector role of ticks, accurate species identification is very important, but it requires time and considerable experience. Among hard ticks (Ixodidae), the genus *Ixodes* contains the most species, which are grouped into subgenera. Species of the subgenus Pholeoixodes belong to a problematic group, as they share morphologic and ecologic characters (Estrada-Peña et al. 2017a, b). In a recent review of available taxonomic literature, Guglielmone et al. (2020) pointed to the problems and importance of correct tick identification, mainly for species of medical, veterinary, and evolutionary importance. For example, a comparative test of tick species identification conducted by a network of European researchers in 14 laboratories specialising in ticks provided an overall misidentification rate of almost 29.6% (Estrada-Peña et al. 2017c), which highlighted the need for molecular methods to identify ticks. DNA barcoding methods serve for accurate and rapid identification of tick species and provide the basis for a molecular data platform for the family Ixodidae (Krčmar et al. 2022). Recently, molecular methods have increasingly been used for accurate tick identification, especially in groups of morphologically very similar species (Hornok et al. 2017, 2021; Krčmar et al. 2022). Thanks to the DNA barcoding method, the occurrence of *I.kaiseri* was recorded in Croatia, Germany, Serbia, Hungary, and Romania (Hornok et al. 2017, 2021; Krčmar et al. 2022), Turkey (Orkun and Karaer 2018), and now Slovakia (this study). Subsequently, Slovakia was added to the list of countries where *I.kaiseri* has been recorded. Moreover, by using DNA barcoding, the identity of *I.canisuga* and *I.hexagonus* was also confirmed.

We hypothesize that *I.kaiseri* has been present in Slovakia but was misidentified because of the variability of morphological characters in species of the subgenus Pholeoixodes. Moreover, due to the endophilic mode of life, only engorged individuals can be collected from hosts and their identification is generally more difficult than unfed ticks. Therefore, further studies of species of the subgenus Pholeoixodes are needed, and morphological identifications in previous studies should be confirmed by molecular methods.
